# Delayed pubarche

**DOI:** 10.1186/s13052-021-01134-0

**Published:** 2021-09-06

**Authors:** Francesco Baldo, Egidio Barbi, Gianluca Tornese

**Affiliations:** 1 Department of Medicine, Surgery and Health Sciences, University of Trieste, Piazzale Europa 1, 34127 Trieste, Italy; 2 Department of Pediatrics, Institute for Maternal and Child Health IRCCS “Burlo Garofolo”, via dell’Istria 65/1, Trieste, Italy

**Keywords:** Absent, Pathological, Adrenarche, Pubarche, Adolescent

## Abstract

In healthy adolescents, delayed pubarche is generally a benign condition that is caused by a physiological discrepancy between gonadarche and adrenarche. In presence of other clinical signs and symptoms, delayed pubarche can be caused by single or multiple hormones deficiency (such as adrenal insufficiency, panhypopituitarism and hypothyroidism) and/or genetic conditions (Turner syndrome, androgen insensitivity syndrome). Exposition to endocrine disruptors has also been described as a possible cause of delay of pubic hair development. Basic blood tests, karyotype and first level imaging studies are helpful in the differential diagnosis.

## Introduction

**Fig. 1:**
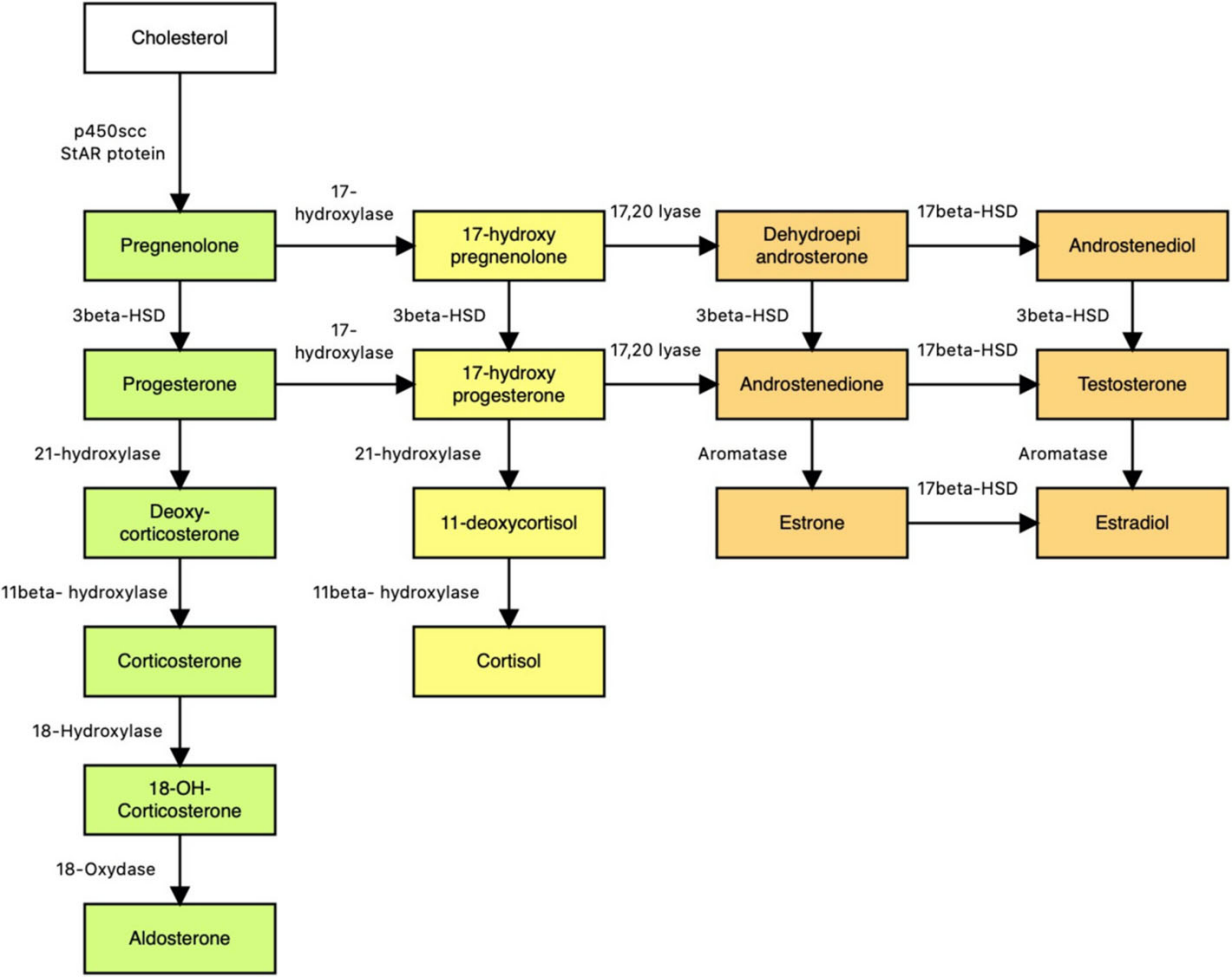
Schematic pathway of adrenal hormones biosynthesis. Color green: mineralocorticoids; color yellow: glucocorticoids; color orange: sexual hormones

Pubarche is a term that indicates the first appearance of pubic hair, which normally develops between 8 and 14 years of age in females, and between 9 and 15 years of age in males. The combination of pubarche and axillarche, which is the first appearance of axillary hair, along with the development of sebaceous and apocrine glands, is called adrenarche [1]. From a biochemical standpoint, adrenarche is caused by a gradual change in the adrenal secretory response to adrenocorticotropic hormone (ACTH). This process starts at around 5 to 6 years of age, well before the appearance of any of the clinical signs just mentioned, and is characterized by a disproportionate rise of 17-ketosteroid in comparison to cortisol, whose production remains stable. This novel secretion pattern results in the production of weak androgens by the zona reticularis of the adrenal cortex, specifically dehydroepiandrosterone (DHEA), which is the most abundant product of the adrenal glands, and its derivatives, dehydroepiandrosterone-sulfate (DHEAS) and androstenedione. While the latter is produced in the adrenal cortex and then converted to testosterone or estrone, DHEAS is the result of an extra-adrenal sulfate reaction to DHEA that happens in the liver, thanks to the sulfate transferase (SULT) enzyme. Apart from ACTH, other factors are thought to influence adrenarche and its clinical manifestations, including the remaining pituitary hormones. Despite the great amount of research on this topic, however, the complexity of these hormonal and biochemical interactions is still unclear and further studies are needed to understand adrenarche’s physiology [2] (Fig. 1).

Although pubarche is strictly connected with puberty and its appearance is considered as one of the typical manifestations of pubertal progression, this event is independent from the gonadic activity of the hypothalamic-pituitary axis. Since pubarche and puberty follows different pathways, they can manifest at different times and, if one of them is pathological, the other can still function normally. Therefore, it is important to not confuse the onset of puberty with the appearance of pubarche, especially in boys [3].

**Fig. 2:**
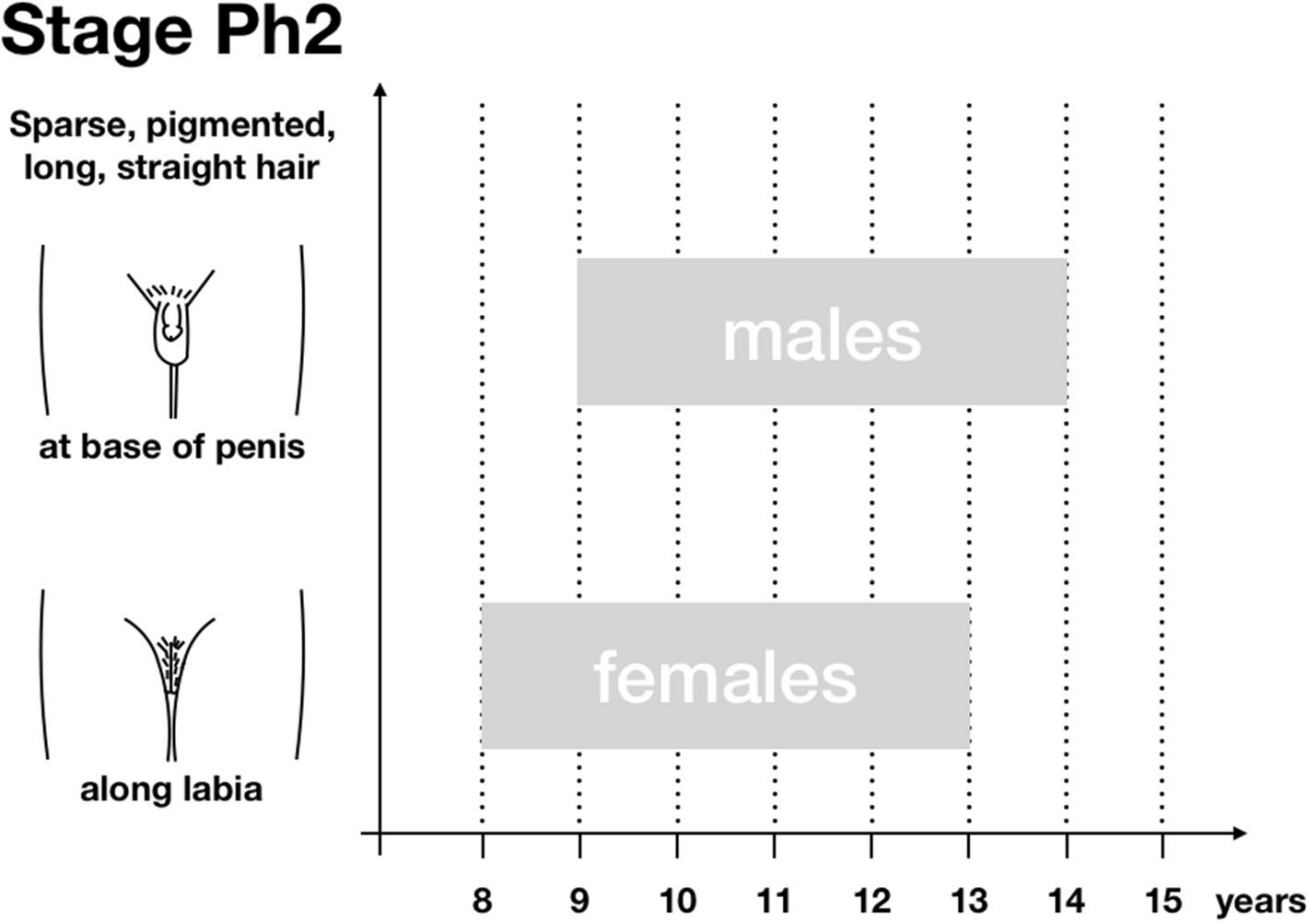
Timing of pubarche in boys and girls

Pubarche is defined premature if it commences before 8 years of age in girls and 9 years of age in boys (which is − 2 standard deviations [SD]). On the other hand, pubarche is considered delayed if it happens after 14 years of age in girls and after 15 years of age in boys (which is + 2 SD) [4] (Fig. 2).

Premature pubarche is a common condition and it is one of most frequent requests for an endocrinologist consultation [5]. Its diagnostic flow chart is well known, and the differential diagnosis includes idiopathic premature pubarche, precocious puberty, congenital adrenal hyperplasia, androgenic tumours, and exogenous androgen exposure [6]. On the other hand, delayed pubarche is scarcely reported in the medical literature, although - by definition - it affects 3% of the population, likewise precocious pubarche. A Pubmed research using the keywords “absent/delayed/late” plus “pubarche/adrenarche” identified 58 articles. Only 7 of these papers were actually covering the topic, none of which extensively or offering a valuable diagnostic approach for clinicians, and 3 of them were case reports [7–13]. We analysed all the papers and divided the subject into two main categories, physiological and pathological pubarche, further discussing the main causes of the latter.

### Physiological delayed pubarche

A discrepancy in the time onset of pubarche and gonadarche, both in males and females, is not infrequent and it is considered as a variation of the normal growth sequence. In males, pubarche usually appears between state G3 and G4 of the genital development. However, the timing of pubic hair appearance is not strict and, basically, a given stage of pubic hair development can be achieved in presence of any stage of genital development. Marshall and Tanner reported that, in a cohort of 228 healthy boys, around 16% of those who already reached stage G4 had not displayed their first pubic hair yet [14]. Since stage G4 was reached at age 13.77 ± 1.02 years, it is expected that at least some males turned 15 without developing pubarche. This range of physiological variability is still partially unexplained and only few involved variables have been identified. For example, Mouritsen et al. have shown that, in young males, specific polymorphisms of enzymes involved in urinary steroid excretion play a role in the timing of pubarche, modulating the interindividual variability of pubic hair appearance [7].

In females, a discrepancy between pubarche and breast development is possible as well. Marshall and Tanner reported that it is not rare to identify girls who reach stage B4 (breast stage 4) before pubic hair appears [15]. Similarly, girls can develop stage P3 or event P4 without any development of the breasts.

Therefore, before considering a delayed pubarche as pathological, a close attention must be given to all the pubertal changes taking place in a child and to their specific timing. As a matter of fact, most of the cases of delayed pubarche in a healthy adolescent with no history and/or signs of a concurrent disease are expected to fall under the physiological range.

### Pathological delayed or absent pubarche

Occasionally, in conjunction with other symptoms or medical signs, a delayed pubarche can be expression of an underlying disease (Table 1).

**Table 1:** Causes and symptoms of pathological delayed pubarche and suggested laboratory testing and imaging

Causes of delayed pubarche	Symptoms	Laboratory testing and imaging
Primary adrenal insufficiency	Fatigue, weakness, weight loss Nausea, vomiting, abdominal pain Hypotension (particularly orthostatic), dehydration Hyperpigmentation Unresponsiveness to intravenous fluid, coma (if severe) Poor feeding and impaired growth (in infants)	Hyponatremia, hyperkalaemia, hypoglycaemia, ketonemia Altered baseline ACTH, cortisol, renin and aldosterone Pathological values of cortisol at ACTH test Search for other autoimmune diseases
Secondary adrenal insufficiency	Fatigue, weakness, weight loss Nausea, vomiting, abdominal pain Impaired growth (GH deficiency)	Altered baseline ACTH and cortisol Normal values of cortisol at ACTH test Search for other pituitary hormones (especially GH) Central nervous system MRI Genetic testing for panhypopituitarism
Androgen insensitivity syndrome	Femininization of external genitalia at birth Undermasculinization and abnormal sexual development Infertility	Karyotype (46, XY) Pelvic ultrasound Genetic testing (AR gene)
Turner syndrome	Physical abnormalities (such as webbed neck, hands and feet lymphedema) Coarctation of aorta Short stature Early loss of ovarian function (primary amenorrhea)	Karyotype (45, X)
Hypothyroidism	Impaired growth Fatigue Constipation Weight gain Sleepiness Dry scalp and skin	Altered TSH, FT4 Search for anti TG and anti TPO antibodies Search for other autoimmune diseases

#### - Primary adrenal insufficiency (Addison’s disease)

Primary adrenal insufficiency is a life-threatening condition caused by impaired secretion of adrenal glucocorticoid and mineralocorticoid hormones [16]. Impaired adrenal steroidogenesis, adrenal dysgenesis and adrenal destruction are the most common causes of primary adrenal insufficiency in children, with congenital adrenal hyperplasia (CAH) and autoimmune destruction being the most frequent [17]. Symptoms of adrenal insufficiency range from insidious to severe and include fatigue, weakness, weight loss, nausea, abdominal pain, vomiting, severe dehydration unresponsive to intravenous fluid, and coma [18]. Infants may also show poor feeding and impaired growth. Hypotension, particularly orthostatic, is usually present at the time of the diagnosis, while hyperpigmentation (due to the hyperproduction of pro-opiomelanocortin) is inconsistent. Laboratory tests show hyponatremia, hyperkalaemia, hypoglycaemia and ketonemia. Baseline levels of ACTH, cortisol, renin and aldosterone, and, more importantly, repetitive pathological values of cortisol during an ACTH stimulation test, are helpful in confirming the diagnosis. Since the appearance of pubic hair is related to the maturation of the adrenal cortex, a delayed pubarche and an adrenarche-gonadarche discrepancy can be expected in adolescents with adrenal insufficiency. In confirmation of this, Hochberg reported three clinical cases of delayed pubarche in Addison’s disease [8, 19]. Therefore, in presence of compatible clinical features (hypotension, hyponatraemic dehydration, hypoglycaemia, hyperpigmentation) or of an history of autoimmune disease (thyroiditis, diabetes, celiac disease), adrenal insufficiency should be carefully considered.

#### - Secondary adrenal insufficiency

Albeit still being unclear, the contribution of ACTH in the regulation of adrenarche has been proved multiple times, so that an absent adrenarche can be indicative of an ACTH deficiency [13, 19]. Secondary adrenal insufficiency is caused by a deficit of pituitary ACTH or hypothalamic corticotropin-releasing hormone (CRH). The clinical presentation is different from the primary form because aldosterone production remains intact, therefore dehydration, hypotension, hyponatremia, and hyperkalaemia are not usually present [20]. Since ACTH is deficient, hyperpigmentation is absent as well. On the other hand, fatigue, weakness, weight loss and gastrointestinal symptoms (nausea, abdominal pain, vomiting) may appear. ACTH deficiency can be isolated or in combination with other pituitary hormones deficiency, in a context of panhypopituitarism [21]. Apart from its congenital form, panhypopituitarism can be caused by any type of damage to the hypothalamus and/or the pituitary gland, including tumours (such as craniopharyngioma), infections, autoimmune diseases, infiltrative diseases, chemotherapy and radiation exposure, and trauma [22]. Since growth hormone (GH)-producing cells are the most sensitive to damage, an isolated deficit of ACTH is very rare. However, since panhypopituitarism can be rather insidious, a growth deflection might not be identified promptly, especially in teenagers after the pubertal spurt.

Two rare variants of anterior panhypopituitarism have been associated with delayed pubarche, even with normal circulating levels of ACTH and cortisol. The first of them is *PIT1/POU1F1* deficiency, a complex pathological entity that typically causes growth hormone, prolactin and thyrotropin deficiency. Taha et al. described 8 patients affected by this condition, all with spontaneous onset and progression of puberty, presenting very low levels of DHEAS and absent or delayed pubic hair appearance [10]. The second condition, called immunoglobulin super family member 1 *(IGSF1)* deficiency, is characterized by central hypothyroidism, delayed surge in testosterone during puberty, macroorchidism and, occasionally, also by hypoprolactinaemia and transient growth hormone deficiency. Van Hulle et al. reported the case of a 19-year-old patient with *IGSF1* that presented delayed adrenarche and pubarche [11].

In both these two genetic conditions, the authors suspect that a reduction of prolactin secretion could be the cause of pubic hair’s delayed appearance, since prolactin receptors are expressed in the adrenal cortex and act synergistically with ACTH in the surge of adrenal androgens [23]. This strict correlation between prolactin and the adrenal gland has been identified also in the opposite scenario, where prolactin is excessively produced. In fact, Tabatabaei et al. described a case of pituitary macroadenoma in which premature pubarche was the first clinical manifestation of the pituitary mass [24].

Therefore, in presence of a delayed pubarche, ACTH should always be tested with a baseline blood sample, along with the remaining pituitary hormones. In presence of panhypopituitarism, genetic consultation and testing should be requested to identify the mutation responsible for the condition. As reported above, in few patients affected by genetic panhypopituitarism, normal ACTH values are not sufficient to guarantee the normal appearance of pubarche, due to the complex interactions between the various pituitary hormones.

#### -Androgen insensitivity syndrome

Androgen insensitivity syndrome (AIS) is a rare condition, with a prevalence that varies from 1/20,000 to 1/100,000 people. It is characterized by a mutation of the androgen receptor *(AR)* gene in the region q11–12 on the X chromosome that leads to an androgen resistance in androgen-dependent tissues. Therefore, subjects with AIS, while having a 46,XY karyotype, typically present feminization of the external genitalia at birth, abnormal sexual development and infertility [25]. The clinical undermasculinization is variable, based on the severity of the androgen sensitivity defect, which can be complete (CAIS), partial (PAIS) or mild (MAIS), with CAIS being the most frequent of the three forms. A pelvic ultrasound must be performed in these patients in order to identify genital abnormalities. Typical findings include the presence of two nondysplastic testes, absent or rudimentary müllerian structures (fallopian tubes, uterus, and cervix) and the presence of a short vagina. Wolffian duct derivatives can be absent or rudimentary, as well as epididymis and vas deferens [26]. AIS subjects normally experience puberty, whose timing is closer to male rather than female standards, and present normal breast development thanks to the aromatization of testosterone to estradiol. Pubarche, on the other, is impaired by androgen resistance, leading to moderate, sparse, or even absent pubic hair, depending on the severity of the genetic defect [27]. DHEAS is typically in range, as in the case reported by Lanciotti et al. [28] Primary amenorrhea is also present, because of the absence of female internal genitalia [29]. Apart for its social and psychological aspect, to make a prompt diagnosis of AIS is critical, since this condition is characterized by an increased, although mild, risk of testicular malignancy, due to the presence of dysgenetic or abdominal testes. Therefore, in females that experience thelarche with absent or delayed adrenarche and primary amenorrhea, pelvic ultrasounds and karyotype should be performed in the suspect of an androgen insensitivity syndrome, whose confirmation is addressed by sequencing the *AR* gene.

#### -Turner syndrome

Turner syndrome (TS) is a common chromosomal disorder (prevalence of 1/2,500 female live births) caused by a total or partial loss of the second X chromosome (genotype 45,X) [30, 31]. TS is characterized by short stature and gonadal dysgenesis, although many other clinical features may appear [32]. In pure gonadal dysgenesis, pubarche is expected to appear regularly, since adrenal androgens are excreted at a normal rate. In fact, in TS patients, pubarche is usually the first developmental feature to appear. In confirmation of this, Bannick et al. reported that the development of pubic hair in TS subjects was similar to that of the normal female population, except for stage 5, that was reached later than healthy girls [33]. In TS, however, the appearance of pubic hair is sometimes delayed or even absent, especially in presence of premature ovarian failure (POF), although adrenarche normally occurs and DHEAS values are within range [9]. A possible explanation of this phenomenon is that ovaries play a role in the adrenal metabolism of DHEA into other androgens during pubarche. Thus, females with POF might require higher levels of androgens than those with normal functioning ovaries [34]. Moreover, in around 20% of TS subjects, pubarche is observed only after estrogen replacement therapy [35]. Turner syndrome diagnosis is sometimes tricky, and it can be delayed even after 18 years of age [36]. Therefore, in presence of a delayed pubarche with compatible TS features (e.g. short stature, primary amenorrhea, dysmorphic features, heart murmur), a karyotype analysis must be performed to rule out this condition.

#### -Hypothyroidism

The relationship between thyroid dysregulation and pubertal development is complex and the studies that explored this matter showed controversial results, especially regarding precocious and delayed puberty [37]. On the other hand, a connection between the activity of the thyroid and the adrenal glands have been reported in various papers, but most of the evidence relies on studies conducted on animals. Huang et al., for example, demonstrated that the administration of thyroxine on immature mice induced the hypertrophy of a fetal part of the adrenal gland that later regresses in adult mice [38]. As Wondiford suggested, it could be inferred that thyroid hormone excess might increase steroid hormone levels also in humans, and especially in children or young adults. Also, by assumption, thyroid hormone deficiency per se might be able to cause a subtle adrenal insufficiency, by acting directly on the gland or by limiting the response to ACTH [39]. Unfortunately, as anticipated, very few papers have examined this topic, but the only two that described a relationship between pubarche and thyroid activity are in line with the concepts described above.

Wilken et al. reported that, in a cohort of 323 of young females, higher prepubertal concentrations of free thyroxine (FT4) were associated with an earlier onset of pubarche, without the appearance of thelarche. This result was independent from common confusing factors, especially obesity [40]. As for delayed pubarche, on the other hand, De Luca et al. reported a dissociation between adrenarche and gonadarche in two longstanding hypothyroid subjects, who presented advanced gonadal development and absent sexual hair, both axillary and pubic. After beginning a substitutive therapy with thyroxine, pubarche finally appeared [41].

#### - Exposition to endocrine disruptors

Phthalates diesters are chemicals with anti-androgenic properties that are used as softeners in plastic production and can be found in various products, such as food packaging, medical devices and building materials. Some studies have showed a relationship between phthalate exposure and changes in girls pubertal timing, but this finding has not been consistently confirmed in the medical literature and the topic remains controversial [42–44]. In a large Danish population-based study, female subjects with higher urinary phthalate concentration had a significant delay of pubic hair development, without alterations of gonadotropins and sex steroid hormone levels [12]. The authors hypothesized that delayed pubarche could be caused by a decrease androgen sensitivity or by a decrease of adrenal steroidogenesis. However, it should be specified that, despite the delayed appearance, statistical analysis showed that vast majority of the girls developed pubic hair within a normal range of age, and it is unclear whether some subjects developed pubic hair after 14 years of age. Further studies are needed to demonstrate if phthalate exposure may induce a pathological delayed pubarche or if they just postpone pubic hair appearance within a physiological, and thus acceptable, range of age.

## Conclusions

In healthy adolescents, delayed pubarche is generally a benign condition that is caused by a physiological discrepancy between gonadarche and adrenarche. In rare occasions, a late appearance of pubic hair can be caused by pathological conditions, most of which are characterized by single or multiple hormones deficiency (such as adrenal insufficiency, panhypopituitarism and hypothyroidism) and/or genetic alterations (Turner syndrome, androgen insensitivity syndrome). In these cases, delayed pubarche is always associated with other clinical signs and symptoms. Basic blood tests are helpful in the differential diagnosis, as well as imaging studies and karyotype. Physicians need to be aware of these conditions to avoid late diagnosis that may worsen patients’ quality of life or even put their life at risk.

## Data Availability

All data generated or analysed during this study are included in this published article and its supplementary information files.

## Abbreviations

ACTH: Adrenocorticotropic hormone
AIS: Androgen insensitivity syndrome
CAH: Congenital adrenal hyperplasia
CAIS: Complete androgen insensitivity syndrome
CRH: Corticotropin-releasing hormone
DHEA: Dehydroepiandrosterone
DHEAS: Dehydroepiandrosterone sulfate
FT4: Free thyroxine
GH: Growth hormone
MAIS: Mild androgen insensitivity syndrome
PAIS: Partial androgen insensitivity syndrome
POF: Premature ovarian failure
SULT: Sulfate transferase
TS: Turner syndrome
